# Soil Microeukaryote Diversity in the Crop Fields of Panama

**DOI:** 10.3390/microorganisms14092124

**Published:** 2026-09-21

**Authors:** Mariel Monrroy, Priscila Moraga-Suazo, Regis Le-Feuvre, José Renán García

**Affiliations:** 1 Research Center in Biochemistry and Applied Chemistry, Faculty of Natural and Exact Sciences, Autonomous University of Chiriqui, David P.O. Box 0427, Panama; 2 Department of Chemistry, Faculty of Natural and Exact Sciences, Autonomous University of Chiriqui, David P.O. Box 0427, Panama; 3 National Research System (SNI), National Secretariat of Science, Technology, and Innovation (SENACYT), Panama City 0816-02852, Panama; 4 Departamento de Ecosistemas y Medio Ambiente, Facultad de Agronomía y Sistemas Naturales, Pontificia Universidad Católica de Chile, Av. Vicuña Mackenna 4860, Santiago 7810000, Chile; priscila.moraga@uc.cl (P.M.-S.); regis.lefeuvre@gmail.com (R.L.-F.); 5 Centro Nacional de Excelencia para la Industria de la Madera (CENAMAD), Pontificia Universidad Católica de Chile, Av. Vicuña Mackenna 4860, Santiago 8320000, Chile

**Keywords:** soil microeukaryotes, 18S rRNA gene metabarcoding, high-throughput sequencing, soil biodiversity

## Abstract

Soil microeukaryotic communities are essential for nutrient cycling and the maintenance of soil fertility, yet their diversity in tropical agricultural systems remains poorly characterized. In this study, high-throughput metabarcoding of the V4 region of the 18S rRNA gene was used to characterize soil microeukaryotic communities across three agricultural regions of Panama. A total of 7714 unique operational taxonomic units (OTUs) were detected in composite soil samples collected from 44 sampling sites. The high-altitude region showed significantly greater species richness and distinct community composition compared with the lowland regions. Principal coordinate analysis identified two major clusters separating the high-altitude and lowland regions. Correlation analyses revealed associations between eukaryotic diversity and soil properties, particularly phosphorus content, electrical conductivity, and terrain slope. Taxonomic assignments, particularly at the species level, should be interpreted cautiously because they are based on 18S rRNA gene metabarcoding and OTU-based classification. These findings contribute to the characterization of soil microeukaryotic diversity in tropical agricultural fields and may support the integration of biodiversity assessments into soil management.

## 1. Introduction

Meeting the growing global demand for agricultural products is becoming one of the world’s most pressing challenges. With food demand projected to increase by 50–60% by 2050, agricultural production may need to almost double to meet future needs [1,2]. At the same time, this increase must be achieved without exacerbating environmental issues such as climate change, biodiversity loss, and the degradation of soil and freshwater resources, which are vital to the long-term development of sustainable agriculture and are increasingly protected by environmental regulations.

Soil quality directly affects crop productivity and is a primary consideration in developing strategies to mitigate and prevent soil erosion. Until recently, soil quality evaluation and management were based almost exclusively on physicochemical characteristics, an approach that oversimplifies the complexity of soil as a natural resource [3]. Soil biological properties and processes remain largely underexplored in the context of soil dynamics and their influence on soil health and quality [4]. Soil microbial communities comprise diverse microorganisms whose activities and interactions contribute to multiple biological processes within the soil environment [5].

In the past decade, research has increasingly focused on advancing our understanding of biological processes within agricultural ecosystems. Despite these advances, further investigation is necessary to better understand the environmental impacts of agriculture, particularly soil degradation and the resulting decline in fertility, which undermine the long-term sustainability of crop production. Understanding microbial dynamics is essential because microbes play important roles in edaphic processes that influence soil fertility [6,7]. An ecological approach has gained attention as a potential means of addressing these issues. This approach considers both the diversity and functional roles of soil communities, has implications that extend beyond basic science, and may support soil restoration through biological processes while reducing reliance on chemical inputs and promoting food and environmental safety.

Culture-independent molecular approaches have greatly expanded the characterization of complex soil communities by enabling the analysis of organisms directly from environmental samples [8,9,10]. Within these communities, soil microeukaryotes comprise a diverse assemblage that includes fungi, protists, algae, and microscopic metazoans such as nematodes. These groups perform diverse ecological functions and occupy different trophic roles in soil ecosystems [11,12].

High-throughput sequencing technologies, together with marker-gene metabarcoding and bioinformatic analyses, have improved the characterization of eukaryotic diversity in complex soil environments [11,12,13]. In particular, 18S rRNA gene metabarcoding is an important culture-independent approach for characterizing the diversity and taxonomic composition of soil eukaryotic communities [13,14]. However, the diversity detected using this approach depends on the selected marker and primers and does not represent a comprehensive assessment of soil eukaryotic diversity. Nevertheless, its application to agricultural soils has enabled the detection of diverse eukaryotic assemblages [14].

Microeukaryotic communities contribute to important ecological processes involved in soil functioning [11]. Their diversity and composition can vary substantially among habitats and environmental conditions [15,16]. Therefore, in this study, we aimed to characterize microeukaryotic communities in three agricultural regions of Chiriquí Province, Panama, using an 18S rRNA gene metabarcoding approach. The selected regions represent areas under intensive agricultural use and tillage practices and are located in different climatic zones with contrasting edaphic characteristics. Specifically, we aimed to characterize and compare the alpha and beta diversity and taxonomic composition of soil microeukaryotic communities among the three agricultural regions and to evaluate their relationships with soil physicochemical characteristics. This study is among the first to apply 18S rRNA gene metabarcoding to assess agricultural soils in Panama, highlighting the potential of this approach to improve our understanding of the relationships between microeukaryotic communities and the physicochemical characteristics of different soil types and thereby inform strategies for agricultural sustainability.

## 2. Materials and Methods

### 2.1. Soil Sampling

**Figure 1:**
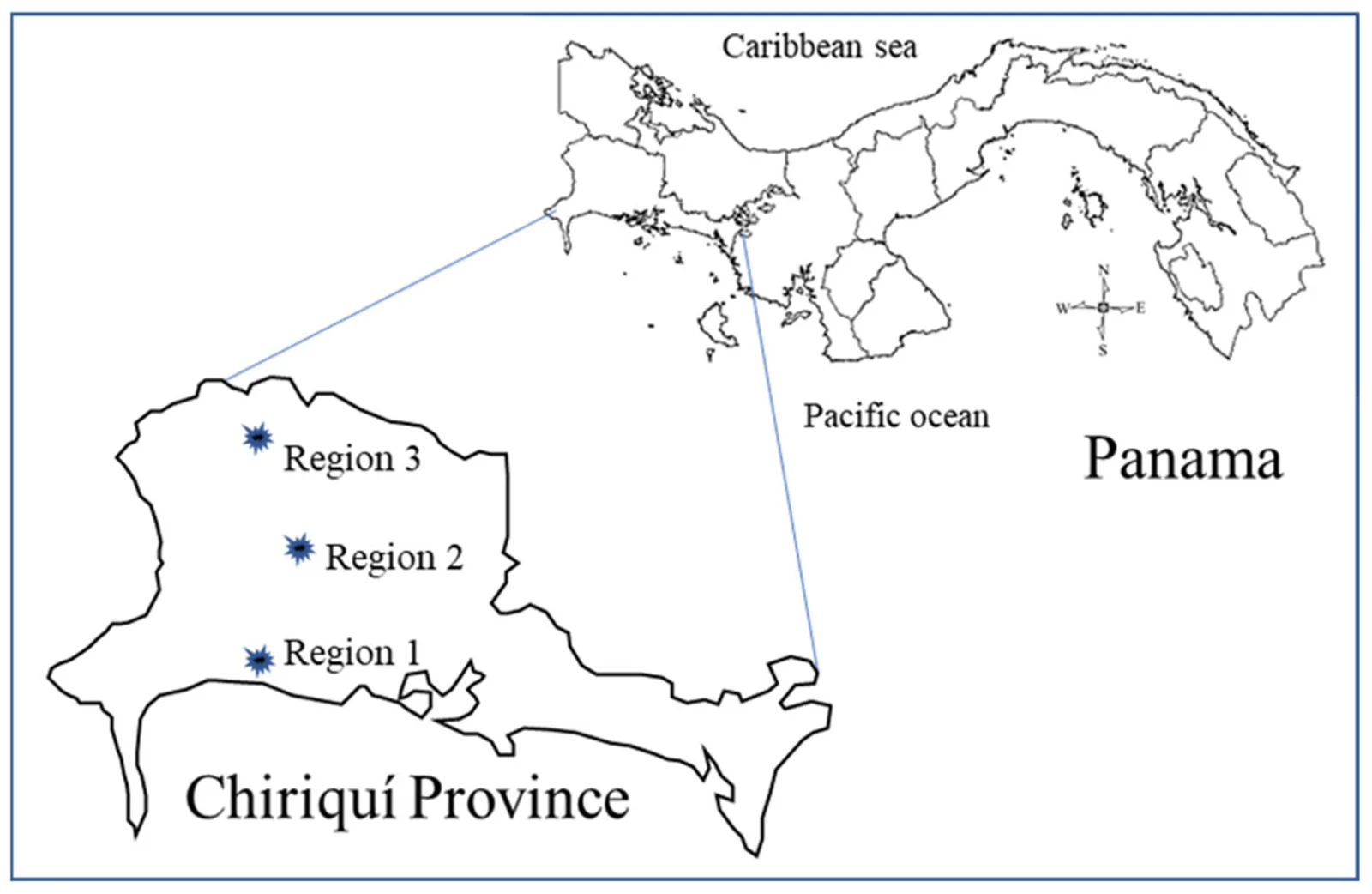
Study regions in Chiriquí Province, Panama.

Soil was collected from farms located in three distinct regions of Chiriqui Province, Panama (Figure 1 and Table 1), each representing different land-use practices. Region 1 comprised lowland irrigated systems primarily used to cultivate rice, maize, and beans on alluvial soils. Region 2 comprised mid-elevation mixed-use systems that integrated seasonal crops such as maize and beans. Region 3 comprised highland horticultural systems in which volcanic soils were intensively cultivated with vegetables such as potatoes, carrots, and leafy greens. Sampling was conducted during the rainy season, between August and October, at 44 farms distributed across Region 1 (*n* = 8), Region 2 (*n* = 3), and Region 3 (*n* = 33). Crop type and crop or field condition at the time of sampling were recorded for each farm (Table 1). Fields encompassed different conditions, including field preparation and recently planted, actively growing, harvest-stage, and post-harvest crops.

At each farm, soil subsamples were collected from the top 20 cm at sampling points spaced approximately 10–20 m apart. The number of subsamples varied among farms, ranging from 4 to 20 (Table 1). Subsamples from each farm were pooled to obtain one composite soil sample per farm. Each composite sample represented an independent farm/site and was treated as a separate statistical unit. Composite samples were then sieved through a 2-mm mesh to remove plant material and other debris. Portions of each composite sample were stored at 4 °C for chemical analysis and at −20 °C for DNA extraction until further use.

### 2.2. Physicochemical Characterization

After sieving, the physicochemical properties of the soil were analyzed. The bulk density of the intact soil was calculated as the dry weight per unit volume. Soil moisture was measured by oven-drying the soil at 105 °C for 24 h. A 1:2 soil-to-water mixture was prepared to measure pH and potassium chloride content using a pH meter (OAKLON PC-700, Singapore). Organic matter (OM) was determined using the Walkley–Black K_2_Cr_2_O_7_–H_2_SO_4_ oxidation method [17]. Soil electrical conductivity was assessed using an aqueous extract of a 1:5 (*v*/*v*) soil-to-water mixture [18]. Soil texture was analyzed using the Bouyoucos method [19]. Available phosphorus content was quantified using the ammonium molybdate–ascorbic acid method [20]. To determine mineral content, soil samples were extracted with nitric acid, and the resulting solution was analyzed using a flame atomic absorption spectrophotometer (AA-7000, Shimadzu, Kyoto, Japan) [20].

### 2.3. DNA Extraction

DNA was extracted from 400 mg of soil per extraction using the DNeasy^®^ PowerSoil^®^ Kit (Qiagen, Hilden, Germany) according to the manufacturer’s instructions. At least five independent extractions were performed for each composite soil sample. A single extract was selected for subsequent analysis based on DNA concentration, purity, and integrity. Total DNA extracted from the soil samples was stored at −20 °C. DNA concentration and purity were assessed using a NanoDrop 2000 spectrophotometer (Thermo Scientific™, Wilmington, DE, USA), and DNA integrity was assessed by electrophoresis on a 0.8% (*w*/*v*) agarose gel at 80 V using a 1-kb DNA ladder (Green BioResearch LLC, Baton Rouge, LA, USA).

Prior to library preparation, Macrogen Inc. (Seoul, Republic of Korea) performed additional quality control using PicoGreen (Invitrogen, Eugene, OR, USA) fluorometry with a Victor 3 fluorometer and gel electrophoresis. DNA concentrations ranged from 30.845 to 106.335 ng/µL, and all samples passed Macrogen’s quality control, which required a DNA concentration of >0.1 ng/µL for MiSeq amplicon library preparation.

### 2.4. Sequence Processing

High-throughput 18S rRNA gene sequencing was performed using an Illumina MiSeq platform. Raw sequencing data from the PCR amplicons were obtained through a commercial sequencing service (Macrogen, Seoul, Republic of Korea). The V4 region of the 18S rRNA gene was amplified using the primers 18S V4F (5′-CCAGCAGCCGCGGTAATTCC-3′) and 18S V4R (5′-ACTTTCGTTCTTGATTAA-3′) [21,22]. PCR amplification and library preparation were performed using Herculase II Fusion DNA Polymerase and the Nextera XT Index Kit V2 (Illumina, San Diego, CA, USA), following the standardized protocol of the commercial sequencing provider. Library quality control showed final library fragment sizes of approximately 557–580 bp. Sequencing was performed on an Illumina MiSeq platform using 301-bp paired-end reads with 30% PhiX.

### 2.5. Bioinformatics Analysis

After sequencing, part of the bioinformatics analysis was performed by Macrogen Inc. (Seoul, Republic of Korea). Paired-end reads generated using the Illumina MiSeq platform were assembled using FLASH (version 1.2.11). Preprocessing and operational taxonomic unit (OTU) clustering were performed using CD-HIT-OTU. The preprocessing workflow included read filtering and trimming, initial clustering of filtered reads at 100% identity using CD-HIT-DUP, identification and removal of chimeric reads, removal of noise sequences, and subsequent clustering of representative nonchimeric sequences. Assembled reads between 350 and 500 bp in length were retained. OTUs were clustered de novo using a 97% sequence similarity cutoff. Taxonomic relative abundances were calculated from quality-filtered OTU counts before rarefaction by expressing the reads assigned to each taxon as a proportion of the total assigned reads within each sample.

Taxonomic assignment was performed using BLAST against the NCBI nucleotide database (NCBI_NT_20200306), whereas diversity analyses were performed using Quantitative Insights Into Microbial Ecology (QIIME 1) [23]. Taxonomic assignments were filtered using minimum thresholds of 85% query coverage and 85% sequence identity. Species-level assignments were interpreted cautiously because of the limited taxonomic resolution of the 18S V4 region. Because the 18S V4 marker broadly targets eukaryotic DNA, all eukaryotic sequences recovered from the soil samples were retained to characterize the overall soil eukaryotic assemblage, including protists, fungi, nematodes, other metazoans, and plant-associated sequences. For visualization of taxonomic composition at the phylum level, non-eukaryotic sequences were excluded, and the relative abundances of the retained eukaryotic taxa were renormalized to 100% within each sample using R (version 4.5.3; R Core Team, Vienna, Austria). Phyla with ≥5% relative abundance in at least one sample were displayed individually, whereas the remaining phyla were grouped as “Others.” Unassigned eukaryotic sequences were retained as a separate category. The relative abundance plot was generated using the ggplot2 package in R.

Prior to alpha-diversity analysis, the OTU table was rarefied to a common sequencing depth of 160,101 reads per sample, corresponding to the minimum sequencing depth among the 44 samples included in the study. Rarefaction was performed in R (version 4.5.3; R Core Team, Vienna, Austria) using the rarefy_even_depth function from the phyloseq package, with random subsampling without replacement. OTU richness, Chao1, Shannon, and Simpson’s diversity index (1 − D) were calculated from the rarefied OTU table. The Shannon index was calculated using base-2 logarithms (log_2_). Good’s coverage and rarefaction curves were used to assess the sufficiency of sequencing depth.

Beta diversity was assessed using principal coordinates analysis (PCoA) and unweighted pair group method with arithmetic mean (UPGMA) clustering based on unweighted pairwise UniFrac distance matrices [24]. PCoA was performed in R (version 4.5.3; R Core Team, Vienna, Austria). Differences in beta diversity among regions were assessed using permutational multivariate analysis of variance (PERMANOVA) with 999 permutations based on the unweighted UniFrac distance matrix. Homogeneity of multivariate dispersion among regions was evaluated using Permutational analysis of multivariate dispersions (PERMDISP). PERMANOVA and PERMDISP analyses were performed in R using the vegan package. UPGMA trees were generated using DendroUPGMA: A dendrogram construction utility [25] and visualized using FigTree (version 1.4.4).

### 2.6. Statistical Analysis

Normality was assessed using standardized skewness and kurtosis, and homogeneity of variances was evaluated using Levene’s test. For variables meeting the normality assumption, differences among regions were evaluated using one-way ANOVA when variances were homogeneous and Welch’s ANOVA when variances were heterogeneous. Moisture, pH, OTU richness, and Chao1 were analyzed using one-way ANOVA followed by Bonferroni-adjusted multiple comparisons, whereas slope, pH (CaCl_2_), and phosphorus were analyzed using Welch’s ANOVA followed by Games–Howell multiple comparisons. OM, electrical conductivity, Shannon diversity, and Simpson diversity did not meet the assumption of normality and were therefore analyzed using the Kruskal–Wallis test. Significant Kruskal–Wallis tests were followed by Bonferroni-adjusted pairwise comparisons. Statistical significance was set at *p* < 0.05.

Spearman’s rank correlation analysis was used to evaluate associations among soil physicochemical properties, OTU richness, and diversity indices because some variables did not meet the assumption of normality. Statistical analyses were performed using Statgraphics Centurion XVIII (Statgraphics Technologies, Inc., The Plains, VA, USA).

Principal component analysis (PCA) was performed in R (version 4.5.3; R Core Team, Vienna, Austria) to explore multivariate patterns among soil physicochemical properties, OTU richness, and diversity indices. Variables were centered and scaled prior to PCA to account for differences in measurement units and magnitudes. Partial least squares discriminant analysis (PLS-DA) was used to evaluate the classification of samples by region. The PLS-DA included 44 samples and 11 predictor variables. Predictor variables were centered and scaled during model training. A two-component PLS-DA model was fitted, and model performance was evaluated using leave-one-out cross-validation (LOOCV). Classification performance was summarized using overall accuracy and a confusion matrix. PLS-DA was performed in R using the caret and pls packages.

## 3. Results

The regions studied were located in different climatic zones and exhibited contrasting edaphic characteristics. Regions 1 and 2 were situated at average altitudes of 12.5 and 367.3 m above sea level, respectively. Both regions had a warm, humid climate, with temperatures generally ranging from 21 to 33 °C and mean annual rainfall of approximately 1673 mm [26]. Region 3 was located at an average altitude of approximately 2107.4 m above sea level and had a temperate-cold mountain climate owing to its high elevation and frequent cloud cover. This region was characterized by a short dry season and a prolonged rainy season. Annual rainfall ranged from approximately 2000 to 3810 mm [27], and the mean annual temperature was 16.4 °C.

Rice, squash, beans, maize, and chili pepper were cultivated in Region 1; pigeon pea and cassava in Region 2; and lettuce, onions, potatoes, celery, carrots, cabbage, and broccoli in Region 3.

### 3.1. Physicochemical Characteristics of Soils

**Figure 2:**
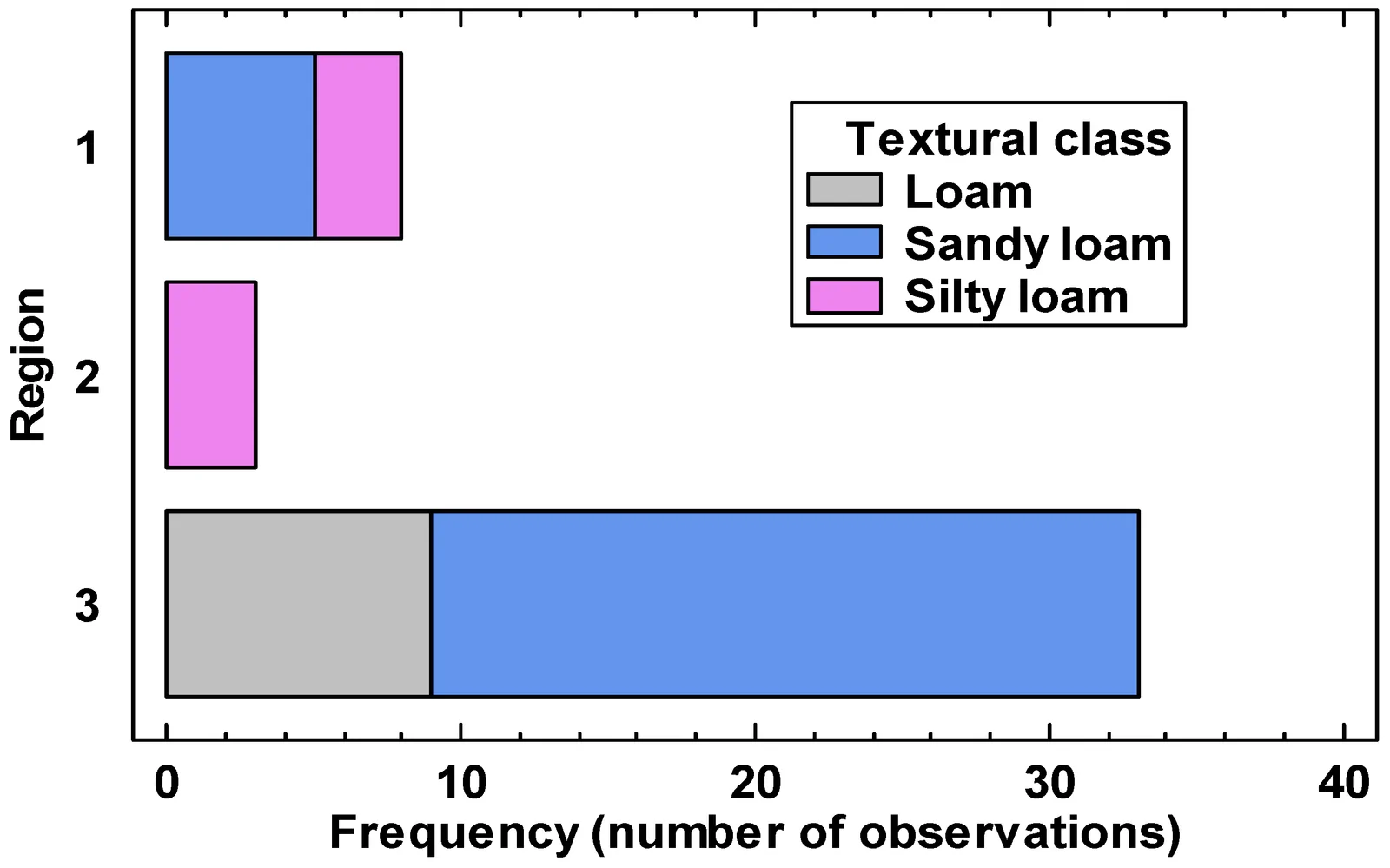
Distribution of soil texture in the three regions.

In Region 1, the soil textures were sandy loam and silty loam, whereas Regions 2 and 3 were characterized predominantly by silty loam and sandy loam, respectively (Figure 2). The physicochemical properties assessed included moisture, OM, pH (H_2_O) and pH (CaCl_2_), electrical conductivity, available phosphorus, and texture (Table 2). At the 95% confidence level, Region 3 exhibited the highest values for electrical conductivity, phosphorus, and slope, with statistically significant differences from the other two regions. Moisture and OM content differed significantly among the three regions, with Region 2 exhibiting the highest values; both pH (H_2_O) and pH (CaCl_2_) differed significantly between Regions 2 and 3.

### 3.2. Data Quality and Diversity

Alpha diversity across all sites was assessed using OTU richness and the Chao1, Shannon, and Simpson diversity indices (Table 3). The Chao1 index, which estimates species richness, indicated that Region 3 had significantly greater estimated richness than Regions 1 and 2 (*p* < 0.05).

A total of 12,634,523 reads were obtained from the 44 soil samples, of which 10,002,809 were retained after quality filtering, with sequencing depths ranging from 160,101 to 288,896 reads per sample. Across the 44 sampling sites, 7714 unique OTUs were detected, of which 5881 were assigned at the species level. Good’s coverage ranged from 99.87% to 99.99%, indicating high sequencing coverage. Rarefaction to 160,101 reads per sample retained all 44 samples for subsequent alpha-diversity analyses. OTU richness was significantly higher in Region 3 soils than in soils from the other regions.

**Figure 3:**
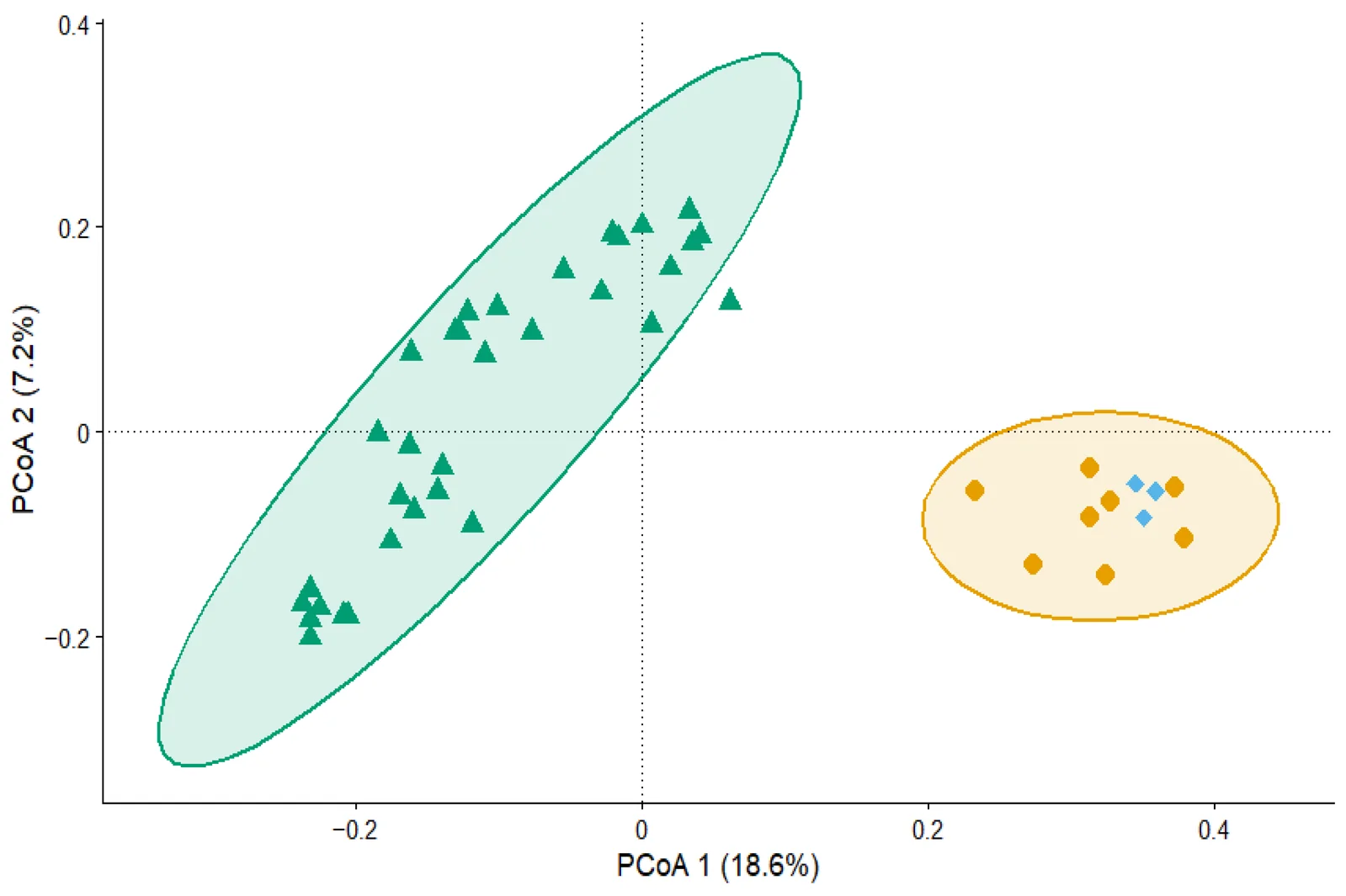
Principal coordinates analysis (PCoA) based on unweighted UniFrac distance matrices. Regions 1 (orange squares), 2 (blue diamonds), and 3 (green triangles) are shown. PCoA1 and PCoA2 explained 18.6% and 7.2% of the total variation, respectively.

**Figure 4:**
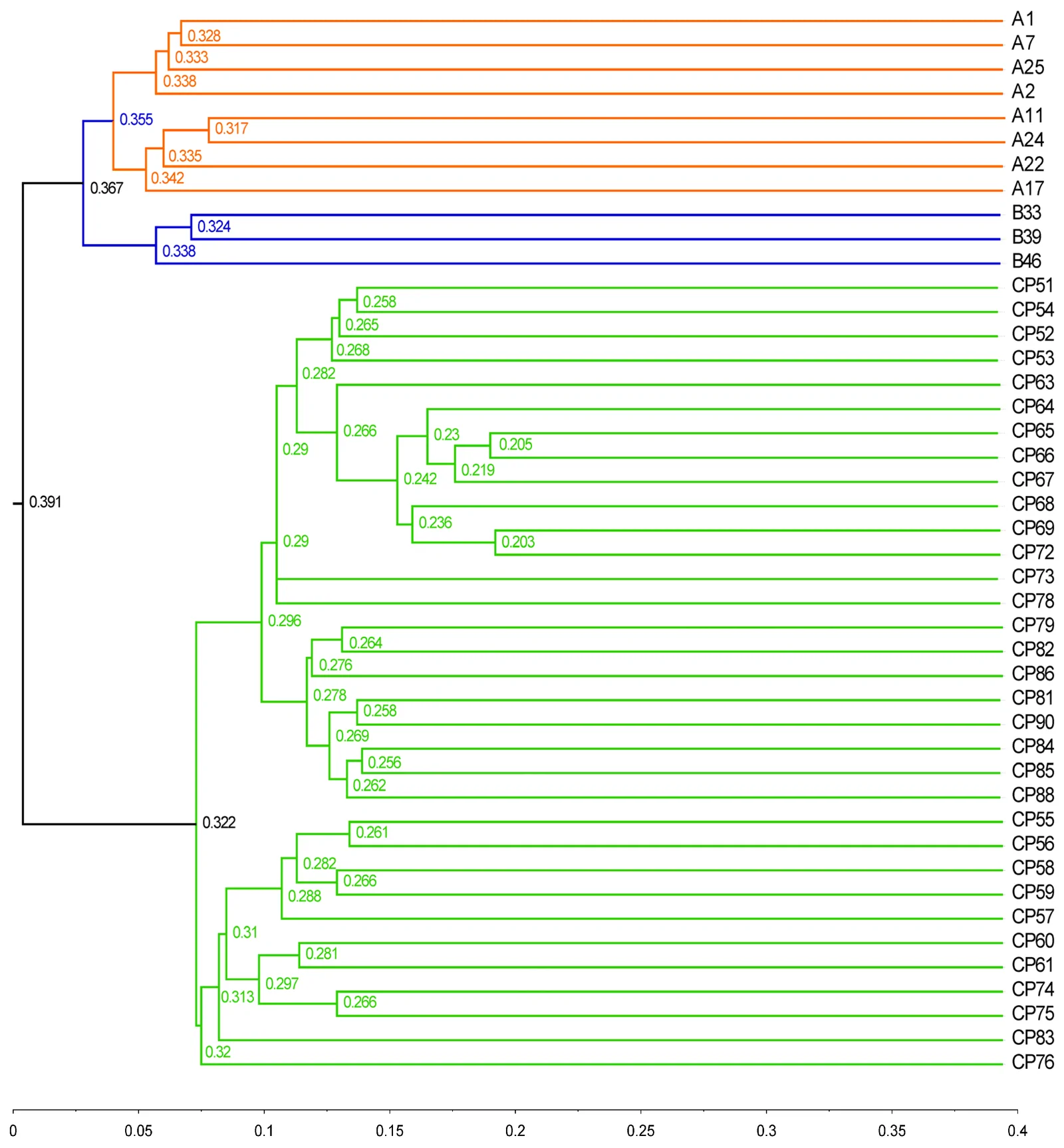
UPGMA clustering based on the unweighted UniFrac distance matrix using average linkage. Branch lengths indicate the distance scale of the dendrogram. Regions 1 (orange), 2 (blue), and 3 (green) are shown.

Beta diversity was assessed using PCoA and UPGMA clustering (Figure 3 and Figure 4). The first two PCoA axes explained 18.6% and 7.2% of the total variation, respectively. PCoA indicated a tendency for Region 3 samples to separate from those of Regions 1 and 2. PERMANOVA indicated significant differences in beta diversity among regions (pseudo-F = 5.28, R^2^ = 0.205, *p* = 0.001). However, PERMDISP was also significant (F = 6.87, *p* = 0.005), indicating that dispersion differences may also contribute to this pattern. UPGMA clustering showed a comparable grouping pattern.

**Figure 5:**
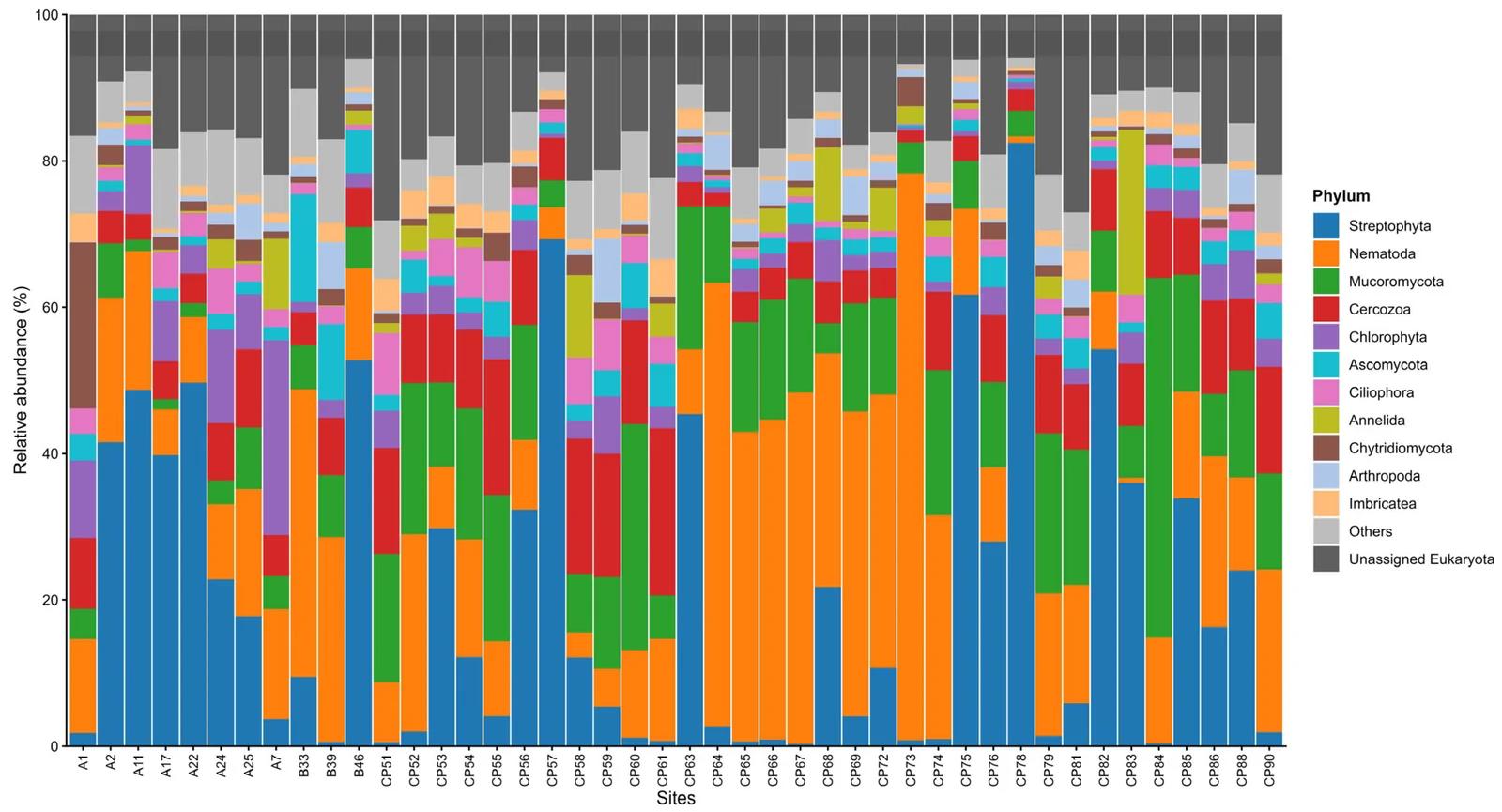
Relative abundance of eukaryotic communities at the phylum level across the 44 soil samples. Phyla with ≥5% relative abundance in at least one sample are shown individually; the remaining phyla are grouped as “Others.” Unassigned eukaryotic sequences are shown separately.

A taxonomic summary of relative abundances (Figure 5, Table 4) showed that soil microeukaryotic community composition varied among the three regions. Region 1 had relatively high abundances (>5%) of *Annelida*, *Cercozoa*, *Chlorophyta*, *Chytridiomycota*, *Mucoromycota*, *Nematoda*, and *Streptophyta*; Region 2 had relatively high abundances of *Ascomycota*, *Cercozoa*, *Mucoromycota*, *Nematoda*, and *Streptophyta*; and Region 3 had relatively high abundances of *Annelida*, *Ascomycota*, *Cercozoa*, *Ciliophora*, *Mucoromycota*, *Nematoda*, and *Streptophyta*.

Despite regional variation, a few phyla had the highest relative abundances: *Streptophyta* in Region 1 (27%), *Nematoda* in Region 2 (25%), and *Nematoda* in Region 3 (20%).

### 3.3. Correlation Between Species Richness, Diversity and the Physicochemical Properties of the Soil

**Figure 6:**
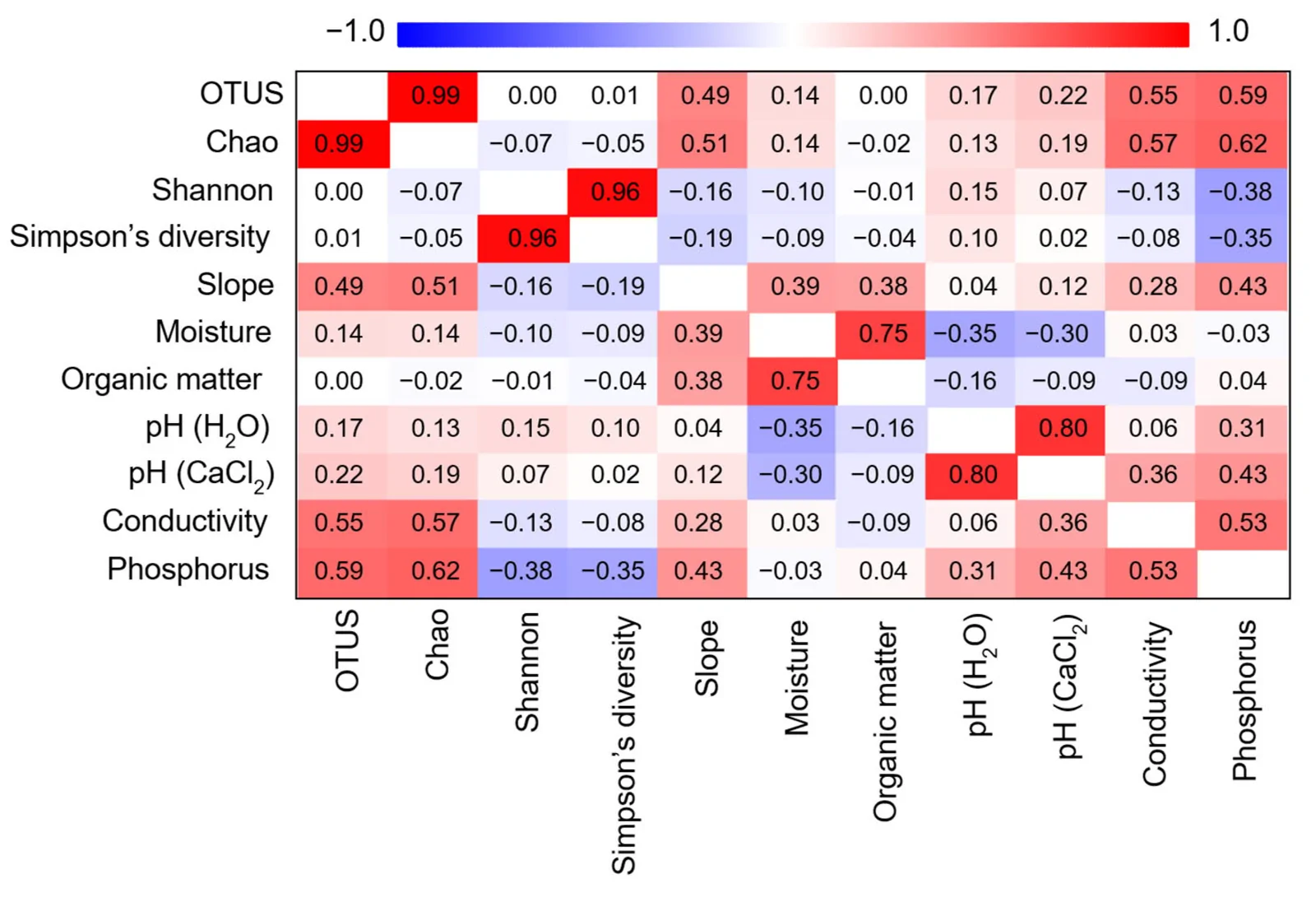
Spearman’s rank correlation matrix for species richness, diversity indices, and soil physicochemical properties. Correlation coefficients are shown for all pairwise relationships.

Figure 6 shows Spearman’s rank correlations between species richness, diversity indices, and soil physicochemical properties. OTU richness was strongly correlated with the Chao1 index (r_s_ = 0.99, *p* < 0.001) and moderately correlated with slope (r_s_ = 0.49, *p* < 0.001), electrical conductivity (r_s_ = 0.55, *p* < 0.001), and phosphorus content (r_s_ = 0.59, *p* < 0.001). The Chao1 index was moderately correlated with slope (r_s_ = 0.51, *p* < 0.001), electrical conductivity (r_s_ = 0.57, *p* < 0.001), and phosphorus content (r_s_ = 0.62, *p* < 0.001). The Shannon index was strongly positively correlated with the Simpson index (r_s_ = 0.96, *p* < 0.001) and weakly negatively correlated with phosphorus content (r_s_ = −0.38, *p* = 0.013). The Simpson index was also weakly negatively correlated with phosphorus content (r_s_ = −0.35, *p* = 0.021). In addition, slope was moderately positively correlated with phosphorus content (r_s_ = 0.43, *p* = 0.004) and weakly positively correlated with moisture (r_s_ = 0.39, *p* = 0.010) and OM (r_s_ = 0.38, *p* = 0.013). Moisture content was strongly positively correlated with OM (r_s_ = 0.75, *p* < 0.001) and weakly negatively correlated with pH (CaCl_2_) (r_s_ = −0.30, *p* = 0.049) and pH (H_2_O) (r_s_ = −0.35, *p* = 0.021). Soil pH (H_2_O) was strongly negatively correlated with pH (CaCl_2_) (r_s_ = −0.80, *p* < 0.001) and weakly positively correlated with phosphorus content (r_s_ = 0.31, *p* = 0.041). Soil pH (CaCl_2_) was weakly positively correlated with electrical conductivity (r_s_ = 0.36, *p* = 0.018) and moderately positively correlated with phosphorus content (r_s_ = 0.43, *p* = 0.040). Electrical conductivity was moderately positively correlated with phosphorus content (r_s_ = 0.53, *p* = 0.021).

### 3.4. Pattern Recognition and Classification Using the Parameters of Richness, Diversity and Soil Physicochemical Properties

**Figure 7:**
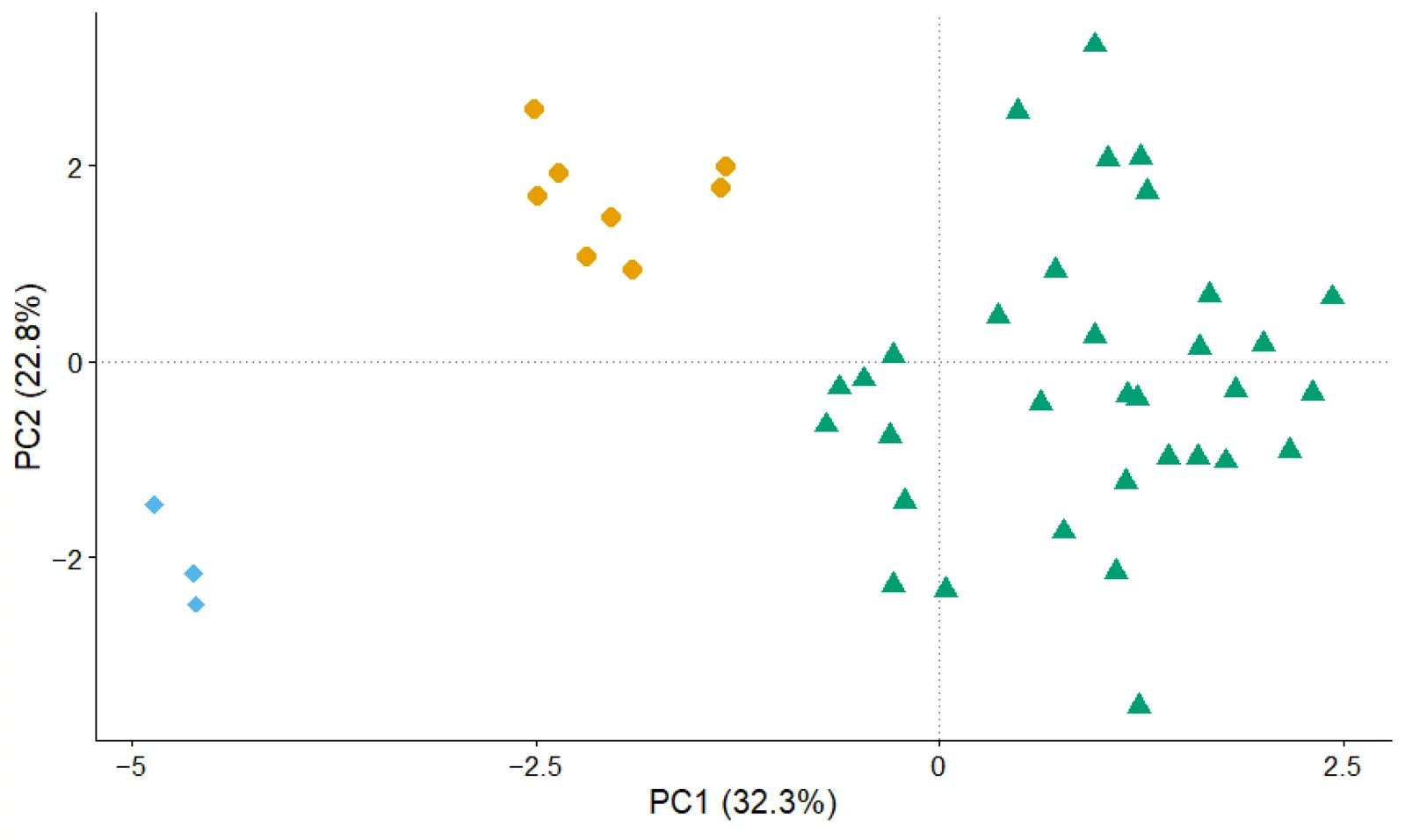
Principal component analysis (PCA) based on richness, diversity, and soil physicochemical properties. PC1 and PC2 explained 32.3% and 22.8% of the total variance, respectively. Regions 1, 2, and 3 are represented by orange circles, blue diamonds, and green triangles, respectively.

PCA was used to identify patterns in soil physicochemical properties and biodiversity indices. The first two principal components showed differentiation among soils from the three regions (Figure 7). The first component explained 32.3% of the total variation and was primarily associated with phosphorus content, the Chao1 index, and OTU richness (loadings = 0.425, 0.451 and 0.447, respectively), whereas the second component explained 22.8% of the total variation and was primarily associated with moisture content and pH (H_2_O) (loadings = −0.470 and 0.410, respectively). The complete loadings for PC1 and PC2 are presented in Table 5. The PCA distribution of the 44 sites suggested some differentiation among regions based on soil properties and biodiversity indices; however, Region 2 was represented by only three samples. Two-component PLS-DA with LOOCV correctly classified 43 of 44 samples (97.7% overall accuracy). All samples from Regions 1 and 2 were correctly classified, whereas 32 of 33 samples from Region 3 were correctly classified (Table 6).

## 4. Discussion

The diversity and composition of soil microeukaryotic communities varied considerably among the agricultural regions studied in Panama. Regions 1 and 2 shared similar low-altitude climatic conditions, whereas Region 3, situated at a higher elevation with a temperate-cold mountain climate, showed distinct community patterns. The differences observed in beta diversity suggest an association between regional variation in soil microeukaryotic community composition and the edaphic characteristics of the studied soils.

According to the USDA Soil Taxonomy [28], the three regions exhibited different edaphic characteristics. Formal soil taxonomic classification was not performed in this study; therefore, potential soil types were inferred only from the measured physicochemical characteristics. The characteristics of soils in Region 1 were consistent with those of Inceptisols, including limited profile development inferred from their texture, pH, and OM content. In Region 2, the high OM content and silty loam texture were consistent with characteristics commonly associated with Mollisols, including relatively high fertility and well-developed surface horizons. Region 3 showed more heterogeneous soil characteristics, with variations in texture and OM content, suggesting potential similarities to either Inceptisols or Mollisols depending on local environmental conditions. This edaphic variability is important for understanding the distribution and function of microeukaryotes within these agricultural ecosystems and their potential contributions to agricultural sustainability. Soil pH, soil type, and electrical conductivity are well-established factors associated with biological activity. Microbial communities play critical roles in soil functioning, and strong relationships exist between soil properties and microbial communities [29].

Alpha-diversity analyses indicated that Region 3 had significantly higher species richness, as reflected by OTU richness and Chao1 estimates, whereas the Shannon and Simpson diversity indices did not differ significantly among regions. This pattern suggests that although species richness was higher in Region 3, overall diversity incorporating both richness and evenness did not differ significantly among regions. Despite being an area of intensive agricultural production, Region 3 had the highest richness, suggesting that factors beyond land-use intensity may be associated with community assembly. One possible explanation is the use of organic fertilizers, particularly poultry manure, which is a common agricultural practice in this region and may increase nutrient availability and soil microhabitat heterogeneity. Such inputs may promote the coexistence of a greater number of taxa by providing additional ecological niches and resources, even under intensive management conditions. Similar patterns have been reported in other agroecosystems, where organic amendments increased microbial richness [30].

Although alpha-diversity analyses revealed differences in species richness, particularly in Region 3, these metrics alone do not fully explain variation in community composition. PERMANOVA indicated significant differences in beta diversity among regions; however, because multivariate dispersion also differed significantly among regions, the PERMANOVA result should be interpreted cautiously.

Beta diversity patterns further indicated differentiation among regions. Both PCoA and UPGMA analyses showed a tendency for Region 3 samples to cluster separately from those of Regions 1 and 2, suggesting that altitude, climate, and soil properties may be associated with these regional patterns. These findings are consistent with previous studies showing that beta diversity is closely associated with climate and habitat structure [31,32].

Taxonomic analyses revealed that a limited number of phyla had relatively high abundances within the assemblages. The relatively high abundance of *Streptophyta* in Region 1 may reflect the influence of surrounding vegetation and plant–root associations; however, because 18S rRNA gene metabarcoding can detect plant-derived DNA, some of these sequences may have originated from roots or plant residues rather than soil microeukaryotic organisms. The relatively high abundance of *Nematoda* in Regions 2 and 3 may be associated with soil disturbance and nutrient enrichment related to agricultural activities. The detection of *Ciliophora* in Region 3 may be associated with high soil moisture and OM inputs characteristic of high-altitude agricultural environments. These patterns are broadly consistent with those reported in previous studies. For example, Ren et al. [33] investigated eukaryotic microbial communities in permafrost soils of the Qinghai–Tibet Plateau and found that groups such as *Nematoda*, *Ciliophora*, *Ascomycota*, *Cercozoa*, *Arthropoda*, and *Basidiomycota* were dominant despite the extreme environmental conditions. Their relative abundances and richness varied markedly among locations, with patterns largely associated with edaphic and climatic conditions. Similarly, Bai et al. [34] reported a high relative abundance of *Streptophyta* in reed rhizosphere soils from a constructed wetland, highlighting the potential ecological relevance of this phylum to plant–soil interactions. Together, these findings suggest that environmental factors (e.g., altitude, moisture, and vegetation) and management practices (e.g., fertilization intensity) may influence the taxonomic composition of soil microeukaryotic communities, consistent with the alpha- and beta-diversity patterns observed in the present study.

In addition to describing community composition, the taxonomic assignments presented in Table 4 provide potentially valuable practical insights. Sequences assigned to taxa such as *Meloidogyne enterolobii* (Region 1) and *Globodera pallida* (Region 3) suggest the potential occurrence of plant-parasitic nematodes of agronomic importance [35,36]. These assignments showed high BLAST sequence similarity (99–100% identity and 100% query coverage); however, 18S rRNA gene metabarcoding alone does not provide definitive species-level identification, which would require targeted molecular or morphological validation. In addition, the detection of saprotrophic fungi (e.g., *Mortierella* spp.) and microalgae (*Chlamydomonas* spp. and *Desmodesmus* spp.) highlights groups associated with soil fertility and nutrient cycling that may have potential as bioindicators of soil health [37,38,39]. Furthermore, sequences assigned to *Oscheius carolinensis*, a species known to be associated with entomopathogenic bacteria [40], suggest potential biotechnological relevance that warrants further investigation. Collectively, these findings suggest that community profiling not only enhances ecological understanding but may also identify taxa of potential interest for sustainable agriculture and biotechnological applications.

Correlation analysis revealed significant associations between eukaryotic diversity metrics and soil phosphorus content, electrical conductivity, and terrain slope. These findings are consistent with previous studies. Köninger et al. [41] reported that plant-available phosphorus was an important factor associated with the richness of protists, tardigrades, and nematodes. Ren et al. [33] demonstrated that alpha diversity and community structure were differentially associated with location and soil properties, particularly phosphorus content. However, our findings differ from those of Shen et al. [15] and Shi et al. [42], who identified soil pH as an important factor associated with eukaryotic microbial distribution. In the present study, no significant correlations were observed between soil pH and the Shannon and Simpson diversity indices.

PCA further showed differentiation among soils from the three regions by integrating physicochemical properties and biodiversity indices within a multivariate framework. The relatively high loadings of phosphorus content, Chao1, and OTU richness on the first principal component indicated their contributions to the observed multivariate variation. The second principal component was primarily associated with soil moisture and pH (H_2_O), suggesting that these variables contributed to an additional dimension of variation among samples. The distribution of sampling sites suggested differentiation among the three regions based on the combination of soil properties and diversity metrics, although Region 2 was represented by only three samples. Similarly, previous studies using 18S rRNA gene metabarcoding have identified phosphorus, pH, and soil moisture as environmental variables associated with variation in microeukaryotic community composition [41,43]. Moreover, two-component PLS-DA with LOOCV correctly classified 43 of 44 samples (97.7%); however, given the small and unbalanced regional sample sizes, particularly the three samples from Region 2, this high classification accuracy should be interpreted cautiously and requires validation in an independent dataset.

These findings highlight associations among environmental gradients, soil properties, and soil microeukaryotic communities across the three agricultural regions studied in Panama. These regional patterns may improve our understanding of soil biodiversity in tropical agricultural systems, although broader studies are needed before extrapolating the findings to other regions.

## 5. Conclusions

Soil microeukaryotic communities varied among the three agricultural regions studied in Panama, which exhibited contrasting edaphic characteristics. Region 3 had significantly higher species richness and showed differences in community composition compared with the lowland regions, suggesting potential associations with altitude, nutrient availability, and soil properties.

Phosphorus content, electrical conductivity, and terrain slope were the main environmental variables associated with patterns of richness and diversity. Multivariate analyses further indicated regional differentiation based on the combination of soil physicochemical properties and biodiversity indices. These findings underscore the importance of considering environmental gradients in soil biodiversity assessments of the agricultural regions studied in Panama. The main limitations of this study include unequal sampling among regions, particularly the small sample size in Region 2, the absence of functional analyses, and the limited resolution of species-level taxonomic assignments based on 18S rRNA gene metabarcoding. Future studies incorporating functional analyses, more balanced regional sampling, targeted validation of species-level assignments, and long-term monitoring will be important for clarifying the ecological roles of soil microeukaryotes and their potential contributions to agricultural productivity and soil health.

## Figures and Tables

**Table 1:** Geographical locations, soil types, and crop or field conditions of the sampled sites.

Region	Site Code	No. Subsamples	Geographical Coordinates	Altitude (m)	Crop/Field Condition at Sampling
1	A1	4	08°25′17.65″ N, 082°43′43.86″ W	15	Field preparation
1	A2	5	08°20′16.47″ N, 082°32′07.54″ W	11	Field preparation
1	A7	7	08°22′06.00″ N, 082°31′45.84″ W	13	Field preparation
1	A11	6	08°21′28.61″ N, 082°31′29.27″ W	10	Maize—actively growing
1	A17	8	08°21′40.86″ N, 082°31′30.24″ W	12	Rice—actively growing
1	A22	7	08°21′51.66″ N, 082°33′13.44″ W	15	Rice—actively growing
1	A24	6	08°21′25.41″ N, 082°31′31.07″ W	9	Chili pepper—actively growing
1	A25	6	08°22′01.38″ N, 082°31′48.48″ W	15	Chili pepper—actively growing
2	B33	6	08°32′52.68″ N, 082°35′33.60″ W	337	Pigeon pea and cassava—actively growing
2	B39	6	08°32′50.88″ N, 082°35′32.94″ W	335	Pigeon pea—actively growing
2	B46	6	08°34′41.10″ N, 082°35′35.46″ W	430	Pigeon pea—actively growing
3	CP51	6	08°50′38.73″ N, 082°33′10.71″ W	2250	Field preparation
3	CP52	16	08°50′47.58″ N, 082°33′02.82″ W	2200	Lettuce—actively growing
3	CP53	6	08°50′59.64″ N, 082°32′52.80″ W	2160	Field preparation
3	CP54	20	08°51′01.26″ N, 082°32′49.74″ W	2155	Field preparation
3	CP55	9	08°51′31.62″ N, 082°35′54.84″ W	1825	Lettuce—recently planted
3	CP56	5	08°51′30.90″ N, 082°35′54.18″ W	1835	Field preparation
3	CP57	6	08°51′23.76″ N, 082°35′59.82″ W	1850	Onion—actively growing
3	CP58	6	08°51′24.48″ N, 082°35′58.62″ W	1837	Field preparation
3	CP59	6	08°51′25.62″ N, 082°35′56.70″ W	1805	Field preparation
3	CP60	7	08°48′28.92″ N, 082°37′29.16″ W	1571	Field preparation
3	CP61	7	08°48′02.16″ N, 082°37′16.80″ W	1557	Field preparation
3	CP63	9	08°49′49.20″ N, 082°33′54.18″ W	2462	Onion—actively growing
3	CP64	7	08°49′49.20″ N, 082°33′52.86″ W	2473	Field preparation
3	CP65	7	08°49′47.70″ N, 082°33′52.98″ W	2480	Potato—recently planted
3	CP66	8	08°49′47.28″ N, 082°33′52.08″ W	2495	Potato—recently planted
3	CP67	7	08°49′45.24″ N, 082°33′52.68″ W	2510	Potato—recently planted
3	CP68	10	08°49′36.96″ N, 082°33′56.40″ W	2581	Potato—at harvest
3	CP69	7	08°49′35.52″ N, 082°34′00.42″ W	2544	Potato—recently planted
3	CP72	7	08°49′37.86″ N, 082°34′00.78″ W	2536	Potato—recently planted
3	CP73	7	08°49′51.00″ N, 082°33′57.12″ W	2445	Celery—recently planted
3	CP74	6	08°49′52.14″ N, 082°36′20.40″ W	1932	Field preparation
3	CP75	6	08°49′53.94″ N, 082°36′18.84″ W	1920	Field preparation
3	CP76	8	08°49′55.92″ N, 082°36′19.14″ W	1890	Cabbage—actively growing
3	CP78	6	08°51′49.92″ N, 082°33′18.48″ W	2167	Cabbage—actively growing
3	CP79	8	08°50′32.76″ N, 082°35′28.08″ W	1984	Carrot—actively growing
3	CP81	8	08°50′15.42″ N, 082°35′06.06″ W	2101	Broccoli—at harvest
3	CP82	11	08°52′06.06″ N, 082°36′24.36″ W	2040	Potato—at harvest
3	CP83	14	08°52′09.06″ N, 082°36′24.54″ W	2028	Field preparation
3	CP84	6	08°51′54.48″ N, 082°36′19.50″ W	1986	Potato—at harvest
3	CP85	7	08°51′56.64″ N, 082°36′18.48″ W	1982	Lettuce—at harvest
3	CP86	6	08°51′53.16″ N, 082°36′16.50″ W	1996	Carrot—recently planted
3	CP88	7	08°51′55.38″ N, 082°36′16.38″ W	1975	Potato—at harvest
3	CP90	10	08°52′04.38″ N, 082°36′16.08″ W	1973	Onion—recently planted

**Table 2:** Physicochemical properties of soils.

Region	Slope	Moisture (%)	Organic Matter (OM) (%)	pH (H_2_O)	pH (CaCl_2_)	Electrical Conductivity (µS/cm)	Phosphorus (mg/kg)
1	0–1.2 ^a^	15.0–22.4 ^a^	2.9–6.4 ^a^	5.4–6.2 ^ab^	4.8–5.3 ^ab^	22.9–81.8 ^a^	6.7–117.1 ^a^
2	0–1.0 ^a^	44.6–45.1 ^b^	21.1–21.7 ^b^	5.1–5.3 ^a^	4.4–4.8 ^a^	50.4–75.0 ^a^	3.3–6.3 ^a^
3	0.4–19.5 ^b^	11.9–34.1 ^c^	2.6–11.3 ^c^	5.0–6.9 ^b^	4.5–6.3 ^b^	60.8–515 ^b^	55.1–578.8 ^b^
*p*-value	<0.0001	<0.0001	<0.0001	0.0064	0.0018	0.0012	<0.0001

Note: Statistical significance was set at *p* < 0.05. Differences among regions were evaluated using one-way ANOVA, Welch’s ANOVA, or the Kruskal–Wallis test, as appropriate. Post hoc comparisons were performed using Bonferroni-adjusted or Games–Howell multiple comparisons, as appropriate. Different letters indicate statistically significant differences among regions.

**Table 3:** Community richness and diversity.

Region	OTUs	Chao1	Shannon	Simpson’s Diversity Index (1 − D)
1	354–886 ^a^	365–901 ^a^	5.1–7.7 ^a^	0.8–1.0 ^a^
2	464–591 ^a^	479–594 ^a^	5.7–7.0 ^a^	0.9–1.0 ^a^
3	742–1420 ^b^	757–1470 ^b^	2.0–7.7 ^a^	0.3–1.0 ^a^
*p*-value	<0.001	<0.001	0.560	0.533

Note: Statistical significance was set at *p* < 0.05. Differences among regions were evaluated using one-way ANOVA or the Kruskal–Wallis test, as appropriate. Bonferroni-adjusted multiple comparisons were performed following significant ANOVA results. Different letters indicate statistically significant differences among regions.

**Table 4:** Taxonomy of eukaryotic communities in the sampled soils.

Region	Phyla (>5%)	Families (>5%)	Taxa (>1%)
Region 1	Annelida	Enchytraeidae, Glossoscolecidae, and Megascolecidae	*Hemienchytraeus* sp. and *Pontoscolex* *spiralis*
	Cercozoa	Cercomonadidae	uncultured Cercozoa
	Chlorophyta	Chlamydomonadaceae and Scenedesmaceae	*Heterochlamydomonas rugosa*, *Desmodesmus komarekii*, *Chlamydomonas* sp., and *Hylodesmus singaporensis*
	Chytridiomycota	Rhizophlyctidaceae	*Rhizophlyctis rosea*
	Mucoromycota	Calcarisporiellaceae and Mortierellaceae	*Jimgerdemannia lactiflua* and *Mortierella wolfii*
	Nematoda	Cephalobidae, Hoplolaimidae, Meloidogynidae, Pratylenchidae, Rhabditidae, Aporcelaimidae, and Qudsianematidae	*Labronema ferox*, *Aporcelaimellus* sp., *Meloidogyne enterolobii*, *Diphterophora obesa*, and *Pratylenchus zeae*
	Streptophyta	Gentianaceae, Rubiaceae, Scrophulariaceae, Lythraceae, and Poaceae	*Duabanga grandiflora*, *environmental* *Bryophyta sequence*, and *Halenia umbellata*
Region 2	Ascomycota		*Archaeorhizomyces borealis*
	Cercozoa	Cercomonadidae	uncultured Cercozoa
	Mucoromycota	Calcarisporiellaceae, Endogonaceae, and Glomeraceae	*Calcarisporiella* sp.
	Nematoda	Alaimidae, Aphelenchidae, Aporcelaimidae, Cephalobidae, Diphtherophoridae, Longidoridae, Meloidogynidae, Trichodoridae, Tylenchidae, and Tylencholaimidae	*Tylolaimophorus typicus*, *Xiphinema krugi*, *Mesorhabditis* sp., *Filenchus misellus*, *Aphelenchus avenae*, *Wilsonema* sp., and *Aporcelaimellus* sp.
	Streptophyta	Arecaceae, Asteraceae, Rubiaceae, and Lythraceae	*Nertera dichondrifolia* and *Senecio* *integerrimus*
Region 3	Annelida	Enchytraeidae	*Mesenchytraeus rhithralis*
	Ascomycota		Unclassified Coniochaetales and *Fusarium oxysporum*
	Cercozoa	Cercomonadidae and Heteromitidae	uncultured Cercozoa and *Heteromita* *globosa*
	Ciliophora	Colpodidae and Gonostomatidae	*Gonostomum kuehnelti* and *Colpoda* sp.
	Mucoromycota	Mortierellaceae	*Mortierella polygonia*
	Nematoda	Aporcelaimidae, Anguinidae, Aphelenchoididae, Cephalobidae, Diplogasteridae, Heteroderidae, and Rhabditidae	*Pelodera teres*, *Cruznema* sp., *Rhabditoides inermiformis*, *Globodera pallida*, *Oscheius* sp., and *Oscheius carolinensis*
	Streptophyta	Asteraceae, Brassicaceae, Bryaceae, Klebsormidiaceae, Gentianaceae, Gesneriaceae, Cyperaceae, Oxalidaceae, and Rubiaceae	*Lepidium densiflorum*

Note: Species-level assignments reported in this table had BLAST sequence identities of 93–100% and 100% query coverage. Taxa, families, and phyla were included independently according to the relative abundance thresholds indicated in the column headings (>1%, >5%, and >5%, respectively). Blank cells indicate that no taxon at the corresponding taxonomic rank met the specified threshold.

**Table 5:** Loadings of the variables included in the principal component analysis (PCA) for the first two principal components.

Variable	PC1 (32.3%)	PC2 (22.8%)
OTU richness	0.447	−0.145
Chao1	0.451	−0.170
Shannon	−0.092	0.349
Simpson’s diversity index	−0.065	0.322
Slope	0.288	−0.281
Moisture	−0.136	−0.470
Organic matter	−0.296	−0.333
pH (H_2_O)	0.263	0.410
pH (CaCl_2_)	0.322	0.317
Electrical conductivity	0.204	−0.141
Phosphorus	0.425	−0.161

Note: Values represent variable loadings on the first two principal components.

**Table 6:** Confusion matrix for PLS-DA classification of samples by region using leave-one-out cross-validation (LOOCV).

Observed	Region 1	Region 2	Region 3
Region 1	8	0	0
Region 2	0	3	0
Region 3	1	0	32

Note: Rows represent observed regions, and columns represent predicted regions. Overall classification accuracy was 97.7% (43/44 samples).

## Data Availability

The raw sequencing reads generated in this study have been deposited in the NCBI Sequence Read Archive (SRA) under BioProject accession number PRJNA1528830.

## Abbreviations

The following abbreviations are used in this manuscript:

OTUs	Operational taxonomic units
PCoA	Principal coordinates analysis
NGS	Next-generation sequencing
OM	Organic matter
QIIME	Quantitative Insights Into Microbial Ecology
UPGMA	Unweighted pair group method with arithmetic mean
ANOVA	Analysis of variance
PCA	Principal component analysis
PLS-DA	Partial least squares discriminant analysis
PERMANOVA	Permutational multivariate analysis of variance
PERMDISP	Permutational analysis of multivariate dispersions

## References

[1] Hunter M.C., Smith R.G., Schipanski M.E., Atwood L.W., Mortensen D.A. (2017). Agriculture in 2050: Recalibrating Targets for Sustainable Intensification. BioScience.

[2] Falcon W.P., Naylor R.L., Shankar N.D. (2022). Rethinking Global Food Demand for 2050. Popul. Dev. Rev..

[3] Lucero M.E., DeBolt S., Unc A., Ruiz-Font A., Reyes L.V., McCulley R.L., Alderman S.C., Dinkins R.D., Barrow J.R., Samac D.A. (2014). Chapter 10Using microbial community interactions within plant microbiomes to advance an evergreen agricultural revolution. Sustainable Agroecosystems in Climate Change Mitigation.

[4] Carbonetto B., Rascovan N., Álvarez R., Mentaberry A., Vázquez M.P. (2014). Structure, Composition and Metagenomic Profile of Soil Microbiomes Associated to Agricultural Land Use and Tillage Systems in Argentine Pampas. PLoS ONE.

[5] Mishra A., Singh L., Singh D. (2023). Unboxing the black box—One step forward to understand the soil microbiome: A systematic review. Microb. Ecol..

[6] Bakker M.G., Schlatter D.C., Otto-Hanson L., Kinkel L.L. (2014). Diffuse symbioses: Roles of plant–plant, plant–microbe and microbe–microbe interactions in structuring the soil microbiome. Mol. Ecol..

[7] Luo J., Guo X., Tao Q., Li J., Liu Y., Du Y., Liu Y., Liang Y., Li T. (2021). Succession of the composition and co-occurrence networks of rhizosphere microbiota is linked to Cd/Zn hyperaccumulation. Soil Biol. Biochem..

[8] Petrosino J.F., Highlander S., Luna R.A., Gibbs R.A., Versalovic J. (2009). Metagenomic pyrosequencing and microbial identification. Clin. Chem..

[9] Alteio L.V., Schulz F., Seshadri R., Varghese N., Rodriguez-Reillo W., Ryan E., Goudeau D., Eichorst S.A., Malmstrom R.R., Bowers R.M. (2020). Complementary Metagenomic Approaches Improve Reconstruction of Microbial Diversity in a Forest Soil. mSystems.

[10] Kirubakaran R., ArulJothi K.N., Revathi S., Shameem N., Parray J.A. (2020). Emerging priorities for microbial metagenome research. Bioresour. Technol. Rep..

[11] Lentendu G., Singer D., Agatha S., Bahram M., Hannula S.E., Helder J., Tedersoo L., Traunspurger W., Geisen S., Lara E. (2025). EukFunc: A Holistic Eukaryotic Functional Reference for Automated Profiling of Soil Eukaryotes. Mol. Ecol. Resour..

[12] Lara E., Singer D., Geisen S. (2022). Discrepancies between prokaryotes and eukaryotes need to be considered in soil DNA-based studies. Environ. Microbiol..

[13] Wang H., Dumack K., Rissi D.V., Finn D.R., Bonkowski M., Tebbe C.C. (2024). Profiling the eukaryotic soil microbiome with differential primers and an antifungal peptide nucleic acid probe (PNA): Implications for diversity assessment. Appl. Soil Ecol..

[14] Mise K., Otsuka S. (2020). Eukaryotic Microbial Communities in Japanese Arable Andisols Investigated by Amplicon Sequencing of 18S rRNA Genes. Microbiol. Resour. Announc..

[15] Shen C., Liang W., Shi Y., Lin X., Zhang H., Wu X., Xie G., Chain P., Grogan P., Chu H. (2014). Contrasting elevational diversity patterns between eukaryotic soil microbes and plants. Ecology.

[16] Hu W., Jin X., Wang Y., He S. (2018). Diversity of eukaryotic micro-organisms and changes in the dominant fungal taxa composition in relationship with soil environment in the Ebinur Lake wetland. Biotechnol. Biotechnol. Equip..

[17] Walkley A.I.B. (1934). An examination of Degtjareff method for determining soil organic matter and a proposed modification of the chromic acid titration method. Soil. Sci..

[18] (2005). Standard Test Methods for Electrical Conductivity and Resistivity of Water.

[19] (1998). Standard Test Method for Particle-Size Analysis of Soils.

[20] Sadzawka A., Carrasco M., Grez R., Mora M., Flores H., Neaman A. (2006). Métodos de análisis de suelos recomendados para los suelos de Chile.

[21] Lee S., Alkathiri B., Lee C.H., Lee H.W., Jeong D.-H., Kim J.Y., Choe S., Lee S.-H. (2025). 18S rRNA gene metabarcoding for investigation of gastrointestinal parasite diversity in great cormorants. Sci. Rep..

[22] Choi J., Park J.S. (2020). Comparative analyses of the V4 and V9 regions of 18S rDNA for the extant eukaryotic community using the Illumina platform. Sci. Rep..

[23] Caporaso J.G., Kuczynski J., Stombaugh J., Bittinger K., Bushman F.D., Costello E.K., Fierer N., Peña A.G., Goodrich J.K., Gordon J.I. (2010). QIIME allows analysis of high-throughput community sequencing data. Nat. Methods.

[24] Kim H., Kim H., Hwang H.S., Kim W. (2017). Metagenomic analysis of the marine coastal invertebrates of South Korea as assessed by Ilumina MiSeq. Anim. Cells Syst..

[25] Garcia-Vallvé S., Palau J., Romeu A. (1999). Horizontal gene transfer in glycosyl hydrolases inferred from codon usage in Escherichia coli and Bacillus subtilis. Mol. Biol. Evol..

[26] IDIAP (2022). Memoria Anual 2022.

[27] Herrera R., Collantes R., Caballero M., Pittí J. (2021). Caracterización de fincas hortícolas en Cerro Punta, Chiriquí, Panamá. Rev. Investig. Altoandinas.

[28] USDA (1999). Soil Taxonomy. A Basic System of Soil Classification for Making and Interpreting Soil Surveys.

[29] Wang B., Adachi Y., Sugiyama S. (2018). Soil productivity and structure of bacterial and fungal communities in unfertilized arable soil. PLoS ONE.

[30] Shu X., He J., Zhou Z., Xia L., Hu Y., Zhang Y., Zhang Y., Luo Y., Chu H., Liu W. (2022). Organic amendments enhance soil microbial diversity, microbial functionality and crop yields: A meta-analysis. Sci. Total Environ..

[31] Yu H., Yang M., Lu Z., Wang W., Yu F., Zhang Y., Yin X., Yu H., Hu J., Deane D.C. (2024). A phylogenetic approach identifies patterns of beta diversity and floristic subregions of the Qinghai-Tibet Plateau. Plant Divers..

[32] Yao L., Jiang B., Jiao J., Wu C. (2023). Environmental Filtering and Dispersal Limitations Driving the Beta Diversity Patterns at Different Scales of Secondary Evergreen Broadleaved Forests in the Suburbs of Hangzhou. Plants.

[33] Ren Z., Ye S., Li H., Huang X., Chen L., Cao S., Chen T. (2023). Biological Interactions and Environmental Influences Shift Microeukaryotes in Permafrost Active Layer Soil Across the Qinghai-Tibet Plateau. Microb. Ecol..

[34] Bai Y., Liang J., Liu R., Hu C., Qu J. (2014). Metagenomic analysis reveals microbial diversity and function in the rhizosphere soil of a constructed wetland. Environ. Technol..

[35] Leuenberger J., Esnault F., Lebas P.L., Fournet S., Cann M.P., Marhadour S., Prodhomme C., Pilet-Nayel M.L., Kerlan M.C. (2025). Identification by GWAS of marker haplotypes relevant to breed potato for Globodera pallida resistance. Theor. Appl. Genet..

[36] Philbrick A.N., Adhikari T.B., Louws F.J., Gorny A.M. (2020). Meloidogyne enterolobii, a Major Threat to Tomato Production: Current Status and Future Prospects for Its Management. Front. Plant Sci..

[37] Ozimek E., Hanaka A. (2021). *Mortierella* Species as the Plant Growth-Promoting Fungi Present in the Agricultural Soils. Agriculture.

[38] Torres M.J., Bellido-Pedraza C.M., Llamas A. (2024). Applications of the Microalgae Chlamydomonas and Its Bacterial Consortia in Detoxification and Bioproduction. Life.

[39] Shin Y.-S., Do J.-M., Noh H.-S., Yoon H.-S. (2025). Exploring the Potential of *Desmodesmus* sp. KNUA231 for Bioenergy and Biofertilizer Applications and Its Adaptability to Environmental Stress. Appl. Sci..

[40] Torres-Barragan A., Suazo A., Buhler W.G., Cardoza Y.J. (2011). Studies on the entomopathogenicity and bacterial associates of the nematode *Oscheius carolinensis*. Biol. Control.

[41] Köninger J., Ballabio C., Panagos P., Jones A., Schmid M.W., Orgiazzi A., Briones M.J.I. (2023). Ecosystem type drives soil eukaryotic diversity and composition in Europe. Glob. Change Biol..

[42] Shi Y., Xu M., Zhao Y., Cheng L., Chu H. (2022). Soil pH Determines the Spatial Distribution, Assembly Processes, and Co-existence Networks of Microeukaryotic Community in Wheat Fields of the North China Plain. Front. Microbiol..

[43] Zhang A., Potapov A.M., Luo R., Zhang Y., Qiang W., Liu B., Pang X. (2025). Protist communities are correlated with abiotic soil factors, but not resources, prey, or predators along a subalpine secondary succession. Geoderma.

